# Data-driven approaches to study the spectral properties of chemical structures

**DOI:** 10.1016/j.heliyon.2024.e37459

**Published:** 2024-09-06

**Authors:** Ibtisam Masmali, Muhammad Faisal Nadeem, Zeeshan Saleem Mufti, Ali Ahmad, Ali N.A. Koam, Haleemah Ghazwani

**Affiliations:** a Department of Mathematics, College of Science, Jazan University, Jazan, 45142, Saudi Arabia; b Department of Mathematics, COMSATS University Islamabad Lahore Campus, Lahore, 54000, Pakistan; c Department of Mathematics and Statistics, The University of Lahore, Lahore, 54000, Pakistan; d Department of Computer Science, College of Engineering and Computer Science, Jazan University, Jazan 45142, Kingdom of Saudi Arabia

**Keywords:** Predictive modeling, Machine learning, Bismuth tri-iodide, Benzene ring, Energy, Data-driven methodologies, Eigenvalues

## Abstract

The molecular energy, which is the sum of all eigenvalues, is crucial in determining the total π-electron energy of conjugated hydrocarbon molecules. We used machine learning techniques to calculate the energy, inertia, nullity, signature, and Estrada index of molecular graphs for bismuth tri-iodide and benzene rings embedded in P-type surfaces within 2D networks. We applied MATLAB to extract the actual eigenvalues from the data and developed general equations for these molecular properties. We then used these equations to estimate the values and compared them to the actual values through graphical analysis. Our results demonstrate the potential of data-driven techniques in predicting molecular properties and enhancing our understanding of spectral theory.

## 1 Introduction

In the continuously developing discipline of chemistry, a comprehensive understanding of molecular energy and spectral characteristics is imperative for predicting the behavior and interactions of various molecules, such as conjugated hydrocarbon molecules. These molecules exhibit unique chemical properties, like increased stability and reactivity, due to the delocalization of π electrons throughout their structure [[1], [2], [3]]. The concept of molecular energy, which is the total sum of all eigenvalues, is central to studying these molecules. This concept is closely linked to spectral theory, a branch of mathematics that examines the relationship between eigenvalues and eigenvectors of linear operators. Spectral theory provides valuable insights into molecular properties and interactions by analyzing and classifying molecular structures based on their energy and spectral characteristics [[4], [5], [6]].

Researchers increasingly adopt interdisciplinary approaches to tackle complex scientific questions as chemistry advances [7]. Data science and machine learning have emerged as powerful tools to revolutionize the study of molecular properties [8]. These techniques provide a robust framework for analyzing extensive and complex datasets, identifying patterns, and building predictive models that significantly improve our understanding of molecular behavior [9,10]. Machine learning, a subset of artificial intelligence, can train algorithms to recognize patterns and relationships within data, leading to predictions or conclusions regarding molecular behavior. These algorithms can be applied to various tasks, such as predicting molecular energy, determining chemical reactivity, and identifying potential drug candidates [[11], [12], [13]].

In spectral theory and molecular energy, data science and machine learning can be used to develop efficient computational methods for estimating molecular properties. By harnessing these tools, researchers can overcome the limitations of traditional approaches, which often depend on time-consuming and labor-intensive experiments. Additionally, these techniques enable the discovery of new relationships and patterns within data, potentially leading to groundbreaking findings and advancements in the field of chemistry [[14], [15], [16]].

The structure of a chemical compound can be represented by a molecular graph, which can be transformed into various matrices by utilizing several graph properties. The atoms of a structure are connected via a bond, which is directed to the adjacency and distance matrices. The polynomial obtained from the adjacency or distance matrix can be considered a structure's signature. The eigenvalue obtained from the polynomial considered the molecular descriptor and was used in the quantitative structure-property/activity relationship.

Graph of a molecule is a mathematical entity defined as G=(V,E), where V is the set of vertices, also called atoms and E is the set of all the edges of the molecule, also called bonds. Usually, in molecular structure, hydrogen atoms are not contemplated. Adjacency matrices $A=\left[a_{ij}\right]$ of molecules are square matrices of order n, and eigenvalues $\lambda_{1},\lambda_{2},\lambda_{3},\ldots ,\lambda_{q-1},\lambda_{q}$ of A are called the eigenvalues of the molecular structure. The absolute sum of all the eigenvalues is known as the energy of a molecular structure G. Mathematically, it is denoted as $E=\sum_{i=1}^{q}\left|\lambda_{i}\right|$. The set consisting of eigenvalues is also named as the spectrum of the graph G. The spectral characteristics of the graph have been expansively investigated. There are many applications of graph theory in the field of chemical graph theory [[17], [18], [19]]. Among the many applications of spectral theory in chemistry, one of them is based on the adjacent equivalence between the eigenvalues of the structure and the molecular orbital energy level of electrons in conjugated hydrocarbons [20,21].

Rank and nullity play a vital role in graph theory and are associated with the area of linear algebra. Rank represents the sum of molecular structures' positive and negative inertia index. The nullity of a structure η(G) is the number of roots having zero value in the characteristic polynomial of A(G) and represents the stability of the molecular structure. If the molecule is stable, closed-shell means its nullity is zero, whereas if it is unstable, it is highly reactive, and open-shell means its nullity is more significant than zero. Every molecule structure can be expressed as square matrices with only 1's and 0's entries p(G), the positive eigenvalues correspond to the positive inertia index, while n(G), the negative eigenvalues correspond to the negative inertia index.

In recent decades, another important concept known as the Estrada index was introduced by Ernesto Estrada and defined as $EE\left(G\right)=\sum_{i=1}^{n}e^{\lambda_{i}}$. Initially, it was applied to quantify the degrees of folding of long-chain molecular structures, particularly proteins. In a continuation of Estrada index's applications, many studies have been done [[22], [23], [24], [25], [26], [27]].

By the motivation of the above mathematical concepts, the structure of the molecule and its optimal properties are measured through these concepts in the current study. For the investigation, two famous molecules, Bismuth tri-iodide, and benzene, are considered due to their huge applications in chemistry, chemical engineering, and other fields of science. We measure the energy and Estrada index of these structures. In addition, we have also calculated the inertia, nullity, and signature of the molecules. This study aims to minimize the error between molecular graphs' exact and estimated values through polynomial curve fitting. We focus on two specific structures: Bismuth Tri-iodide (BiI3) and a benzene ring embedded in a p-type surface. By comparing the exact and estimated energy and Estrada index for each structure, we aim to demonstrate the effectiveness of our methodology. To achieve this, we employ a multi-step computational process using various software tools.

## 2 Brief description of Bismuth Tri-iodide (BiI_3_)

Bismuth tri-iodide (BiI_3_) is an inorganic structure that is produced by the chemical reactions of iodine and bismuth; this motivated the interest of qualitative studies [28]. BiI_3_ is extremely helpful in subjective inorganic investigations. It was experimentally shown that Bi-doped glass optical strands are among the most capable energetic laser media. Various types of Bi-doped fiber strands are formed and depleted to make Bi-doped fiber lasers and optical loudspeakers [29]. BiI_3_ is a structure consisting of three layers, such that a bismuth atom is packed in between iodide particles to form a repeated I−Bi−I plane [30]. Each monolayer unit of BiI_3_ is stacked with each other via Vander Walls forces [31]. This structure provides ideal 2D material for photovoltaic cells, optoelectronics, and ambient temperature X-rays/gamma rays detectors [32]. These stacking patterns and interlayer distance affect the electronic structure and stability [33]. These electronic properties are modified by intercalation, chemical doping, and mechanical strength [34,35]. Particularly, the optical properties are significantly affected by the interlayer distance. The BiI_3_ forms the material for photodetectors with excellent durability and stability with different bending strains, making them suitable for flexible devices such as for optoelectronics with advanced technologies like optical fiber communication, flexible imaging technologies, complex environmental monitoring, and wearable light sensors [36]. In addition, BiI_3_ also gains attention in gamma-ray detectors or radiographic imaging owing to its strong photon inhibition power due to its high density (5.78 g/cm3), large band gap, and greater effective atomic number. These important characteristics are essential for huge resolutions of room-temperature gamma-ray spectroscopies [37,38]. The tremendous properties of BiI_3_ also lead to the tactile applications of smart sensors, photovoltaic cells, human-machine interfacing and photonics. Through the modification of the morphologies the BiI_3_ can convert into single and twin plates [39].

### 2.1 Computational methodology for molecular graph analysis

**Fig. 1:**
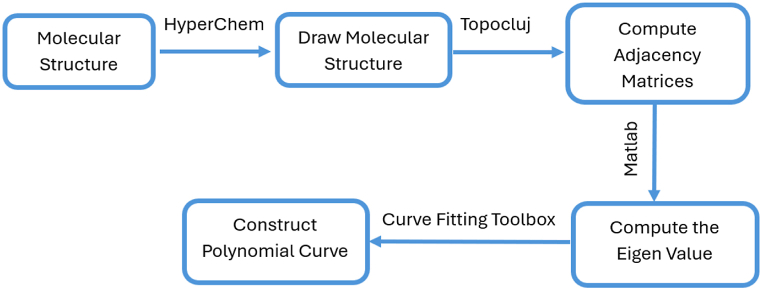
Procedure to calculate the energy and Estrada index of molecular structures.

To measure the energy and Estrada index, different types of computation work are done by different software as shown in Fig. 1.

**Fig. 2:**
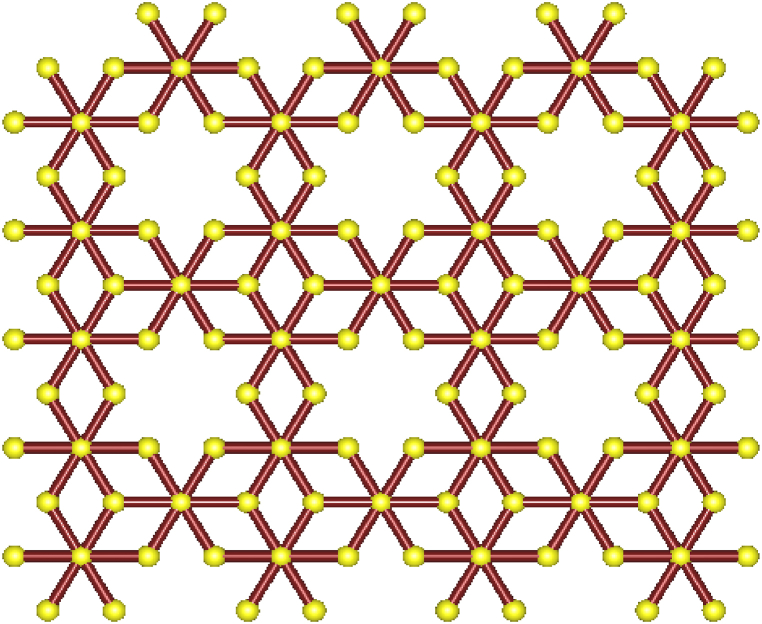
Bismuth tri-iodide.

Following the procedure outlined in Fig. 1, we first used *HyperChem* to draw the molecular structure of BiI_3_, as depicted in Fig. 2.

Secondly, an adjacency matrix of the molecular graph is constructed by using TopoCluj. Third, the matrix's eigenvalues are calculated with Matlab's help. Finally, a polynomial curve of degree two is built through the eigenvalues attained from the adjacency matrix of the molecular structure using cf Toolbox in Matlab.

### 2.2 Energy and Estrada index of BiI_3_

The 2 s-order polynomials which display the energy and Estrada index of the Bismuth Tri-iodide molecule are given by Eqs. (1), (2) respectively.

(1) $$E\left(B\right)=7\times 10^{-9}m^{2}n^{2}-9\times 10^{-5}n^{2}m+0.0003n^{2}-0.0003nm^{2}+13.794nm+9.5506n-0.0014m^{2}+5.1553m-0.3239$$

(2) $$EE\left(B\right)=3.167m^{2}n^{2}-15.839n^{2}m+19.009n^{2}-12.67nm^{2}+110.86nm-44.392n+9.49m^{2}-32.462m+57.857$$

Where m is horizontal and n is vertical unit cells of BiI_3_.

Eqs. (1), (2) can further be written in the form of coefficients as shown in Eqs. (3), (4):

(3) $$E\left(B\right)=\left\{ \begin{array}{cc} n^{2}: & 7\times 10^{-9}m^{2}-9\times 10^{-5}m+0.0003\\ n: & -0.0003m^{2}+13.794m+9.5506\\ 1: & -0.0014m^{2}+5.1553m-0.3239 \end{array}\right.$$

(4) $$EE\left(B\right)=\left\{ \begin{array}{cc} n^{2}: & 3.167m^{2}-15.839m+19.009\\ n: & -12.67m^{2}+110.86m-44.392\\ 1: & 9.49m^{2}-32.462m+57.857 \end{array}\right.$$

The numerical results for the energy of and Estrada index of $BiI_{3}$ through Eqs (1), (2) are calculated at different values of m in Table 1.

**Table 1:** The quadratic equations of the energy and Estrada index of BiI_3_.

(m,n)	Energy(E(B))	EstradaIndex(EE(B))
(1,n)	$2.10\times 10^{-4}n^{2}+23.34n+4.83$	$6.342n^{2}-176.20n+34.88$
(2,n)	$1.28\times 10^{-4}n^{2}+19.86n+9.98$	$0.009n^{2}-103.35n+30.91$
(3,n)	$3.00\times 10^{-5}n^{2}+50.92n+15.12$	$-99.98n^{2}+244.16n-154.05$
(4,n)	$-5.00\times 10^{-5}n^{2}+64.72n+20.27$	$-293.64n^{2}+866.33n-520.03$
(5,n)	$-1.49\times 10^{-4}n^{2}+78.51n+25.44$	$-580.96n^{2}+1763.16n-1067.02$
(6,n)	$-2.39\times 10^{-4}n^{2}+92.30n+30.55$	$-961.94n^{2}+2934.65n-1795.01$
(7,n)	$-3.29\times 10^{-4}n^{2}+106.12n+35.69$	$-1436.58n^{2}+4380.80n-2704.01$
(8,n)	$-4.19\times 10^{-4}n^{2}+119.92n+40.82$	$-2004.89n^{2}+6101.61n-3794.02$
(9,n)	$-5.09\times 10^{-4}n^{2}+133.72n+45.96$	$-2666.85n^{2}+8097.08n-5065.03$
(10,n)	$-5.99\times 10^{-4}n^{2}+147.52n+51.08$	$-3422.48n^{2}+10367.21n-6517.05$

**Fig. 3:**
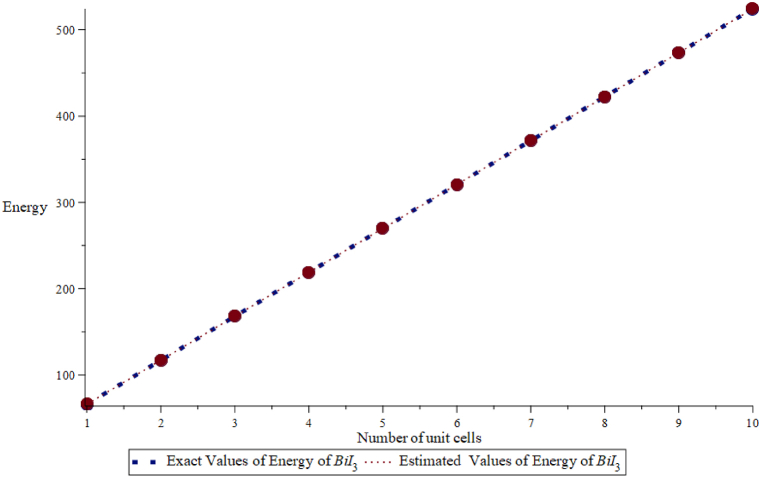
Comparison of exact and estimated values of energy of BiI_3_.

**Fig. 4:**
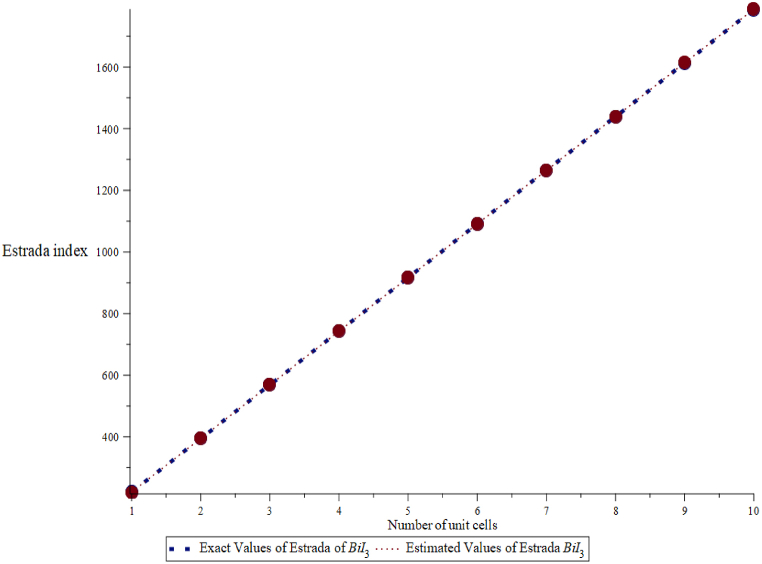
Exact and estimated comparison of Estrada index of BiI_3_.

Further, estimated values of energy and Estrada are computed at different numbers of unit cells by using Eqs. (1), (2) and compared with exact values of energy and Estrada index obtained through constructing the adjacency matrix, as shown in Fig. 3, Fig. 4.

Here, exact values are denoted by a blue dotted line and estimated values by an orange line. These figures show a good agreement between exact and estimated values. For the close view, we also calculate the Mean Absolute percentage error between these values in Table 2, Table 3. The relative error is important because it gives us a valuation of the accuracy of calculations or projections. This allows us to analyze the method we use to identify areas for potential improvements.

**Table 2:** The exact values $E_{ext}\left(B\right)$ and estimated values $E_{est}\left(B\right)$ of the energy of BiI_3_.

(m,n)	$E_{ext}\left(B\right)$	$E_{est}\left(B\right)$	Error	Absolute Error	Absolute Percentage Error	Relative Error (%)
(3,1)	66.950	66.966	0.016	0.016	0.023	$\frac{0.016}{66.966}\times 100\%$ = 0.02 %
(3,2)	116.924	116.989	0.065	0.065	0.056	$\frac{0.065}{116.989}\times 100\%$ = 0.05 %
(3,3)	167.849	167.919	0.070	0.070	0.041	$\frac{0.070}{167.919}\times 100\%$ = 0.04 %
(3,4)	218.775	218.849	0.074	0.074	0.033	$\frac{0.074}{218.849}\times 100\%$ = 0.03 %
(3,5)	269.701	269.779	0.078	0.078	0.029	$\frac{0.078}{269.779}\times 100\%$ = 0.03 %
(3,6)	320.626	320.709	0.082	0.082	0.026	$\frac{0.082}{320.709}\times 100\%$ = 0.03 %
(3,7)	371.552	371.640	0.088	0.088	0.024	$\frac{0.088}{371.640}\times 100\%$ = 0.02 %
(3,8)	422.478	422.570	0.091	0.091	0.022	$\frac{0.091}{422.570}\times 100\%$ = 0.02 %
(3,9)	473.403	473.500	0.097	0.097	0.020	$\frac{0.097}{473.500}\times 100\%$ = 0.02 %
(3,10)	524.328	524.431	0.102	0.102	0.019	$\frac{0.102}{524.431}\times 100\%$ = 0.02 %

Mean Absolute Percentage Error (MAPE) 0.029.

**Table 3:** Exact values $EE_{ext}\left(B\right)$ and estimated values $EE_{est}\;\left(B\right)$ of the Estrada index of BiI_3_.

(m,n)	$E_{ext}\left(B\right)$	$E_{est}\left(B\right)$	Error	Absolute Error	Absolute Percentage Error	Relative Error (%)
(3,1)	215.519	215.515	−0.004	0.004	0.0018	$\frac{-0.004}{215.519}\times 100\%$ = - 0.002 %
(3,2)	427.787	427.780	−0.007	0.007	0.0016	$\frac{-\;0.007}{427.787}\times 100\%$ = - 0.002 %
(3,3)	640.066	640.054	−0.012	0.012	0.0018	$\frac{-\;0.012}{640.066}\times 100\%$ = - 0.002 %
(3,4)	852.338	852.341	−0.002	0.002	0.0002	$\frac{-\;0.002}{852.338}\times 100\%$ = - 0.0002 %
(3,5)	1064.611	1064.634	−0.023	0.023	0.0021	$\frac{-\;0.023}{1064.611}\times 100\%$ = - 0.002 %
(3,6)	1276.885	1276.940	−0.055	0.055	0.0043	$\frac{-\;0.055}{1276.885}\times 100\%$ = - 0.004 %
(3,7)	1489.158	1489.256	0.098	0.098	0.0066	$\frac{0.098}{1489.158}\times 100\%$ = 0.007 %
(3,8)	1701.432	1701.583	0.151	0.151	0.0089	$\frac{0.151}{1701.432}\times 100\%$ = 0.009 %
(3,9)	1919.705	1913.920	0.215	0.215	0.0112	$\frac{0.215}{1919.705}\times 100\%$ = 0.01 %
(3,10)	2125.979	2126.267	0.288	0.288	0.013	$\frac{0.288}{2125.979}\times 100\%$ = 0.01 %

Mean Absolute Percentage Error (MAPE) 0.0343.

**Fig. 5:**
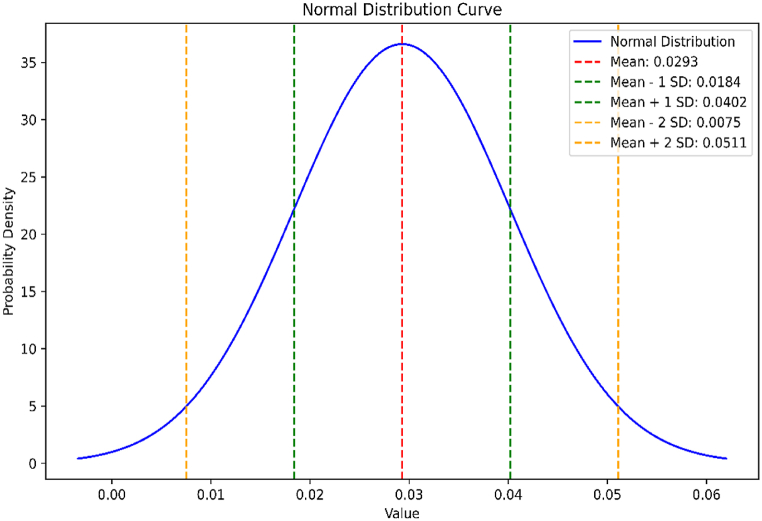
Normal distribution curve for the absolute errors in energy estimation of BiI_3_.

Another way to support this study is to use another statistical method in which we first find the mean absolute error and the standard deviation of the errors across all data points to understand the overall accuracy of the estimation method. The average absolute error for the above data is 0.0763, and the standard deviation is 0.02423519. The normal distribution curve of the data given in Table 2 is shown in Fig. 5.

**Fig. 6:**
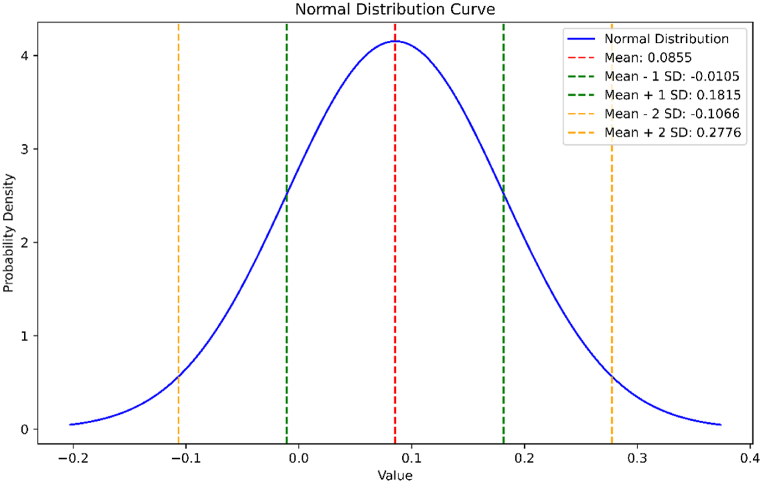
Normal distribution curve for the absolute errors in Estrada index estimation of BiI_3_.

The average absolute error for the above data is 0.0855 and the standard deviation is 0.09604087671403254. The normal distribution curve of the data given in Table 3 is shown in Fig. 6.

In Tables 2 and it is noticed that the exact/actual values of the energy of BiI_3_ are smaller than the value of energy of $BiI_{3}$ obtained from the quadratic equations, i.e. $E_{ext}\left(B\right)<E_{est}\left(B\right)\text{,}$ where, we denote $E_{ext}$ exact and $E_{est}$ the estimated values of energy. Moreover, errors are positive among these values of energy, i.e. Error > 0. Similarly, in Table 3, we have noted that actual values of Estrada index of $BiI_{3}$ are always more than the estimated values, i.e. $EE_{ext}\left(B\right)>EE_{est}\left(B\right)\text{,}$ where, $EE_{ext}$ and $EE_{est}$ represents the exact and estimated value of Estrada index, respectively. We find that the mean absolute percentage error of energy of BiI_3_ is 0.035 and mean absolute percentage error of Estrada index of BiI_3_ is 0.00515. This error analysis shows that the relative errors are generally very small, indicating that the estimated values $EE_{est}\left(B\right)$ are quite close to the exact values $EE_{ext}\left(B\right)$. This suggests that the estimation method is accurate for this dataset, with errors typically less than 0.1 % relative to the exact values.

### 2.3 The inertia, nullity and signature of BiI_3_

This section analyzes the molecular structure of BiI_3_ and its stability through optimal properties. For this, we calculate the numerical results of inertia, nullity and signature of BiI_3_ in Table 4. In Table 4, p(B) shows the positive inertia index whereas n(B) represents the negative inertia index. When the vertical unit cells n are increased at constant m horizontal unit cells, it is found the balance between positive and negative inertia indexes. The difference between positive and negative eigenvalues is called the signature s(B) and found no difference due to balanced behavior of inertia indexes. The results for nullity η(B) are obtained by accounting the eigenvalues having zero value in the characteristic polynomial. The nullity of BiI_3_ is increased with increasing of vertical unit cells of structure as shown in Table 4.

**Table 4:** The inertia, nullity and signature of BiI_3_.

(m,n)	p(B)	n(B)	η(B)	s(B)
(3,1)	16	16	40	0
(3,2)	26	26	60	0
(3,3)	36	36	80	0
(3,4)	46	46	100	0
(3,5)	56	56	120	0
(3,6)	66	66	140	0
(3,7)	76	76	160	0
(3,8)	86	86	180	0
(3,9)	96	96	200	0
(3,10)	106	106	220	0
(3,11)	116	116	240	0
(3,12)	126	126	260	0

## 3 Benzene Ring Embedded (BRE) in P-type-surface

P-type networks are embeddings of sp^2^ carbons in triply periodic surfaces with the same regularity of single-node simple cubic Bravis tilings. It linked among the 230 symmetry classes of Euclidean space. In these embeddings, the edges of the structure are without crossings, and it splits the space into two disjoint regions. A molecular structure consists of entirely $sp^{2}$ atoms, is embeddable in a triply periodic surface, with nonpositive Gaussian arc. These types of carbon structures are called Schwarzites. Schwarzites have exceptional electronic, magnetic, and optical characteristics. The Shwarzites, which are embedded in P-type surfaces, decorate the Bravais lattice in three-dimensional Euclidean space. P-type surfaces can be filled with various coverings of polygons having more sides than hexagons, which are required to create the negative Gaussian curvature.

### 3.1 Energy and Estrada index of BRE

**Fig. 7:**
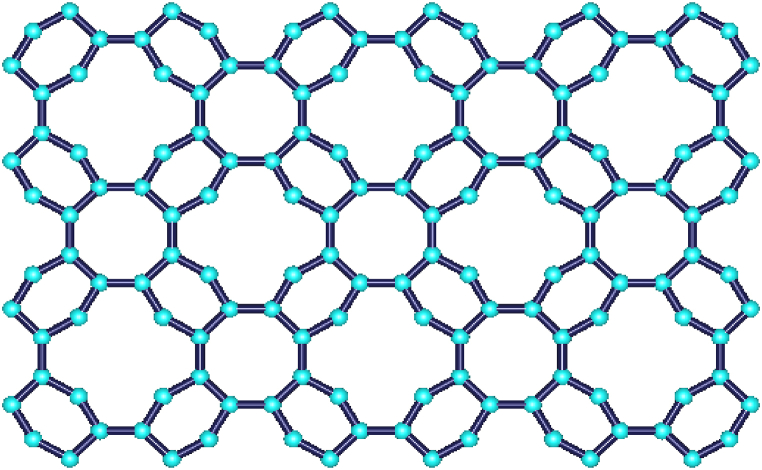
Benzene ring embedded in P-type surface.

The same procedure is followed to measure the energy and Estrada index of BRE as for BiI3. The structure of BRE is constructed through HyperChem, as shown in Fig. 7.

The results of energy and Estrada index in the form of 2 s-order polynomials are shown in Eqs. (5), (6) respectively.

(5) $$E\left(BRE\right)=2\times 10^{-7}m^{2}n^{2}-2\times 10^{-5}n^{2}m+2\times 10^{-5}n^{2}-0.01nm^{2}+35.2nm+0.98n-0.0085m^{2}+0.9773m-0.1176$$

(6) $$EE\left(BRE\right)=0.0542m^{2}n^{2}-0.1387n^{2}m-0.0665n^{2}-0.0145nm^{2}+70.062nm+2.193n-0.1985m^{2}+2.3475m-1.994$$

Eqs. (5), (6) can further be written in the form of coefficients as shown in Eqs. (7), (8):

(7) $$E\left(BRE\right)=\left\{ \begin{array}{cc} n^{2}: & 2\times 10^{-7}m^{2}-2\times 10^{-5}m+2\times 10^{-5}\\ n: & -0.01m^{2}+35.2m+0.98\\ 1: & -0.0085m^{2}+0.9773m-0.1176 \end{array}\right.$$

(8) $$EE\left(BRE\right)=\left\{ \begin{array}{cc} n^{2}: & 0.0542m^{2}-0.1387m-0.0665\\ n: & -0.0145m^{2}+70.062m+2.193\\ 1: & -0.1985m^{2}+2.3475m-1.994 \end{array}\right.$$

The numerical set of results for the energy and Estrada index at different values of m by using Eqs. (5), (6) are shown in following Table 5.

**Table 5:** The quadratic curves for the Energy and Estrada index of BRE.

(m,n)	Energy(E(BRE))	Estradaindex(EE(BRE))
(1,n)	$\frac{1}{5000000}\;n^{2}+36.17\;n+0.8682$	$-0.1510\;n^{2}+72.2405\;n+0.1550$
(2,n)	$-\frac{3}{156250}\;n^{2}+71.34\;n+1.8710$	$-0.1271\;n^{2}+142.2590\;n+1.9070$
(3,n)	$-\frac{191}{5000000}\;n^{2}+106.49\;n+2.8908$	$0.0052\;n^{2}+212.2485\;n+3.2620$
(4,n)	$-\frac{71}{1250000}\;n^{2}+141.62\;n+3.9276$	$0.2459\;n^{2}+282.2090\;n+4.2200$
(5,n)	$-\frac{3}{40000}n^{2}+176.73\;n+4.9814$	$0.5950\;n^{2}+352.1405\;n+4.7810$
(6,n)	$-\frac{29}{312500}n^{2}+211.82\;n+6.0522$	$1.0525\;n^{2}+422.0430\;n+4.9450$
(7,n)	$-\frac{551}{5000000}\;n^{2}+246.89\;n+7.1400$	$1.6184\;n^{2}+491.9165\;n+4.7120$
(8,n)	$-\frac{159}{1250000}\;n^{2}+281.94\;n+8.2448$	$2.2927\;n^{2}+561.7610\;n+4.0820$
(9,n)	$-\frac{719}{5000000}\;n^{2}+316.97\;n+9.3666$	$3.0754\;n^{2}+631.5765\;n+3.0550$
(10,n)	$-\frac{1}{6250}\;n^{2}+351.98\;n+10.5054$	$3.9665\;n^{2}+701.3630\;n+1.6310$

**Fig. 8:**
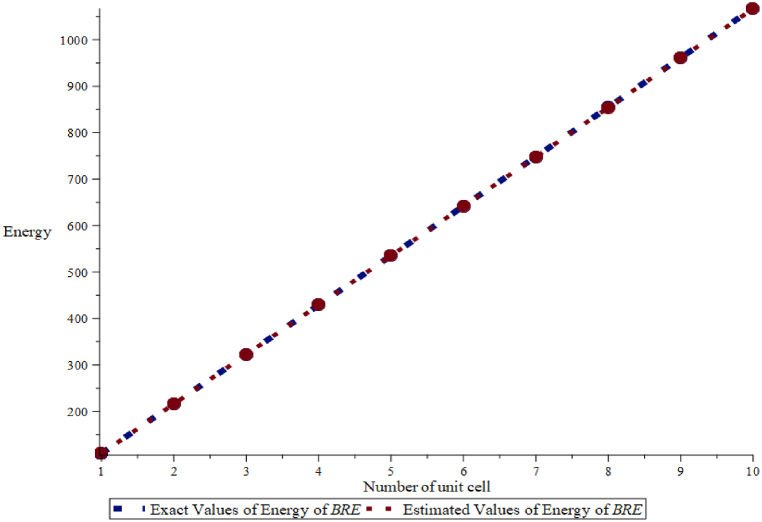
Exact and estimated comparison of energy of BRE.

**Fig. 9:**
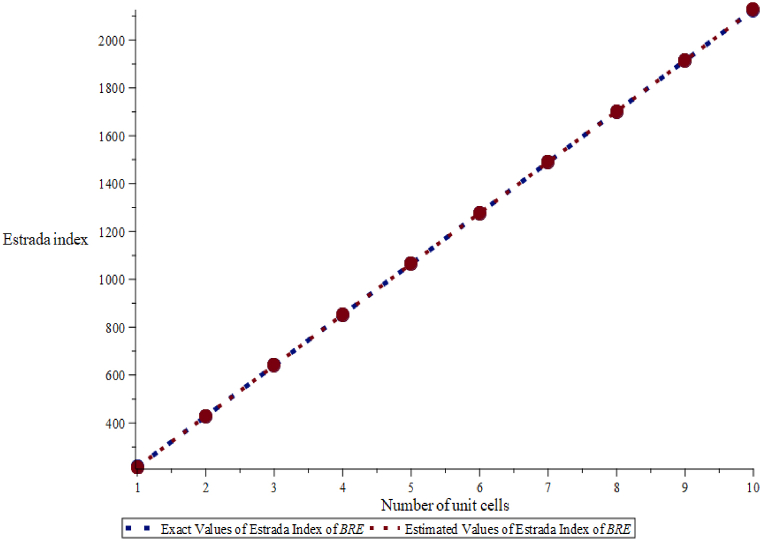
Exact and estimated comparison of Estrada index of BRE.

To check the accuracy of the result, we have compared the estimated values of energy and Estrada index which are obtained from Eqs. (5), (6) with the exact values of energy and Estrada index in Fig. 8, Fig. 9 and found a good agreement between the results.

Here, exact values are denoted by a blue dotted line and estimated values by an orange line. In addition, we calculate the mean absolute percentage error between these values in Table 6, Table 7.

**Table 6:** The exact values $E_{ext}\left(BRE\right)$ and estimated values $E_{est}\left(BRE\right)$ of the energy for BRE.

(m,n)	$E_{ext\;}\left(B\right)$	$E_{est\;}\left(B\right)$	Error	Absolute Error	Absolute Percentage Error	Relative Error (%)
(3,1)	109.376	109.378	0.0014	0.0014	0.0013	$\frac{0.0014}{109.376}\times 100\%$ = 0.001 %
(3,2)	215.887	215.890	0.0036	0.0036	0.0017	$\frac{\;0.0036}{215.887}\times 100\%$ = 0.002 %
(3,3)	322.361	322.360	−0.0010	0.0010	0.0003	$\frac{-\;0.010}{322.361}\times 100\%$ = - 0.003 %
(3,4)	428.834	428.825	−0.0087	0.0087	0.0020	$\frac{-\;0.0087}{428.834}\times 100\%$ = - 0.002 %
(3,5)	535.305	535.170	−0.1347	0.1347	0.0252	$\frac{-\;0.1347}{535.305}\times 100\%$ = - 0.025 %
(3,6)	641.779	641.651	−0.2677	0.2677	0.0417	$\frac{-\;0.2677}{641.779}\times 100\%$ = - 0.04 %
(3,7)	748.264	748.809	−0.4554	0.4554	0.0609	$\frac{-\;0.4554}{748.264}\times 100\%$ = 0.06 %
(3,8)	854.742	854.063	−0.6790	0.6790	0.0794	$\frac{-\;0.6790}{854.742}\times 100\%$ = - 0.08 %
(3,9)	961.220	961.275	−0.9456	0.9456	0.0983	$\frac{-\;0.9456}{961.220}\times 100\%$ = - 0.09 %
(3,10)	1067.699	1067.444	1.2551	1.2551	0.1175	$\frac{1.2551}{1067.699}\times 100\%$ = 0.01 %

Mean Absolute Percentage Error (MAPE) 0.0232.

**Table 7:** The exact values $EE_{ext}\left(BRE\right)$ and estimated values $EE_{est}\left(BRE\right)$ of the Estrada Index for BRE.

(m,n)	$E_{ext\;}\left(B\right)$	$E_{est\;}\left(B\right)$	Error	Absolute Error	Absolute Percentage Error	Relative Error (%)
(3,1)	215.519	215.515	−0.004	0.004	0.0019	$\frac{-\;0.004}{215.519}\times 100\%$ = - 0.002 %
(3,2)	427.787	427.780	−0.007	0.007	0.0016	$\frac{-\;0.007}{427.787}\times 100\%$ = - 0.002 %
(3,3)	640.066	640.054	−0.012	0.012	0.0019	$\frac{-\;0.012}{640.066}\times 100\%$ = - 0.002 %
(3,4)	852.338	852.341	−0.002	0.002	0.0002	$\frac{-\;0.002}{852.338}\times 100\%$ = - 0.0002 %
(3,5)	1064.611	1064.634	0.023	0.023	0.0022	$\frac{0.023}{1064.611}\times 100\%$ = 0.002 %
(3,6)	1276.885	1276.940	−0.055	0.055	0.0043	$\frac{-\;0.055}{1276.885}\times 100\%$ = - 0.004 %
(3,7)	1489.158	1489.256	0.098	0.098	0.0066	$\frac{0.098}{1489.158}\times 100\%$ = 0.007 %
(3,8)	1701.432	1701.583	0.151	0.151	0.0089	$\frac{-\;0.6790}{854.742}\times 100\%$ = - 0.08 %
(3,9)	1913.705	1913.920	0.215	0.215	0.0112	$\frac{\;0.215}{1913.705}\times 100\%$ = 0.01 %
(3,10)	2125.979	2125.267	0.288	0.288	0.0135	$\frac{0.288}{2125.979}\times 100\%$ = 0.01 %

Mean Absolute Percentage Error (MAPE) 0.00723.

**Fig. 10:**
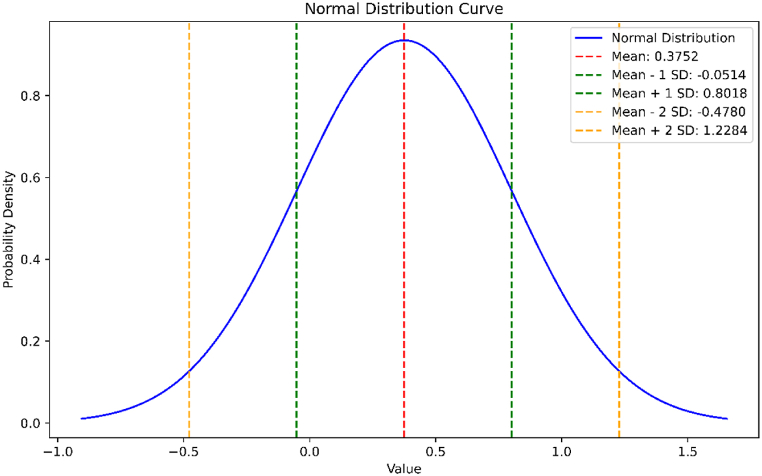
Normal distribution curve for the absolute errors in energy estimation of *BRE*.

The average absolute error for the above data is 0.3761 and the standard deviation is 0.42581697. The normal distribution curve of the data given above is shown in Fig. 10.

**Fig. 11:**
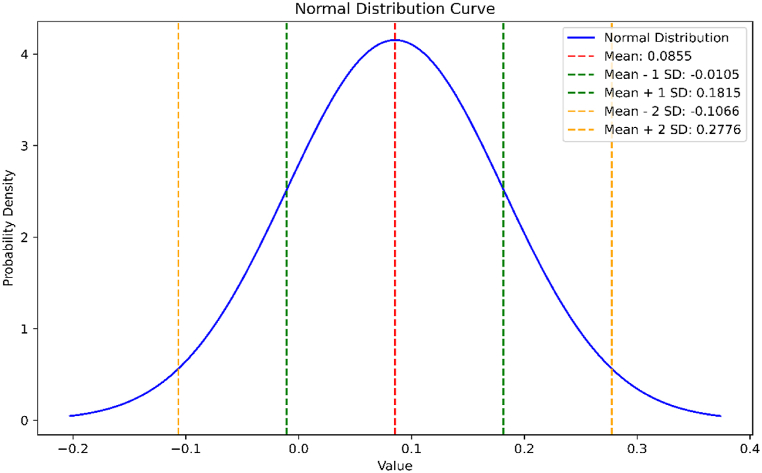
Normal distribution curve for the absolute errors in Estrada index estimation of *BRE*.

The average absolute error for the above data is 0.0855 and the standard deviation is 0.09604087671403254. The normal distribution curve of the data is shown in Fig. 11.

We have observed that the exact value of the energy of BRE is less than first two terms, the gradual increase from the estimated values of energy of BRE is seen. Similarly, exact value of Estrada index of BRE is less than the estimated values of Estrada index of BRE, for few terms, which later have a sudden change in behavior for the remaining five values. We find that the mean absolute percentage error of energy of BRE is 0.04283 and mean absolute percentage error of Estrada index of BRE is 0.00523.

### 3.2 The inertia, nullity and signature of *BRE*

The molecular structure of *BRE* and its stability are analysis through the numerical results of inertia, nullity and signature in Table 8. In Table 8, p(BRE) and n(BRE) shows the positive and the negative inertia indexes and found a balance between results. The signature of molecular structure is displayed by s(BRE) and found the zero values because of the balance behavior of inertia indexes. In the results of nullity denoted by η(BRE), found a constant behavior at all unit cells of the structure as shown in Table 8.

**Table 8:** The inertia, nullity and signature of BRE.

(m,n)	p(BRE)	n(BRE)	η(BRE)	s(BRE)
(3,1)	39	39	2	0
(3,2)	76	76	2	0
(3,3)	113	113	2	0
(3,4)	150	150	2	0
(3,5)	187	187	2	0
(3,6)	224	224	2	0
(3,7)	261	261	2	0
(3,8)	298	298	2	0
(3,9)	335	335	2	0
(3,10)	372	372	2	0
(3,11)	409	409	2	0
(3,12)	446	446	2	0

## 4 Conclusion

We studied the energy, Estrada index, inertia, nullity, signature for BiI_3_ and BRE in current investigation. The following inequalities $E_{ext}\left(B\right)>E_{est}\left(B\right)\text{,}$ $EE_{ext}\left(B\right)>EE_{est}\left(B\right)\text{,}$ $E_{ext}\left(BRE\right)>E_{est}\left(BRE\right)$ and $EE_{ext}\left(BRE\right)>EE_{est}\left(BRE\right)$ have been observed between exact and estimated values of energy of BiI_3_ and BRE. In addition, since the nullity of BiI_3_ and BRE is zero so the molecule of these structures is stable and closed shell.

The numerical values of the energy told us about the correlation between the bond energy of π-electrons and every orbital in π-electrons correspond to the each eigen value of the graph under consideration. If we calculate the energy for a single unit, such as (1,1), we obtain a positive integer. As we increase the order horizontally, for example, moving from (1,1) to (1,2), (1,3), (1,4) and so on, the energy of each subsequent unit will also be increased. This is because the number of vertices grows, and consequently, the order of the adjacency matrix also increases. Similarly, we proceeded vertically, for example, from (1,1) to (2,1), (3,1), and so on. As the order increased, handling the calculations became increasingly difficult. Therefore, we generalized our graph and applied statistical methods to analyze the general behavior of the data. To determine the energy of a particular unit, we generated a polynomial of a certain order to estimate the energy of the desired unit. The positive eigenvalues were linked with the antibonding level, negative eigenvalues were linked with bonding levels, and zero eigenvalues were associated with the nonbonding level.

By employing MATLAB to extract actual eigenvalues from the data and generate general equations, we aimed to bridge the gap between the actual and estimated values of these molecular properties. We used the Mean Absolute Percentage Error (MAPE) as the standard metric for error in Table 2, Table 3, Table 6, Table 7. When the MAPE between actual and expected values was close to zero, it generally indicated that the model's predictions were accurate on average. Additionally, we observed that the energy of BiI_3_ and BRE was 0.035 and 0.04, respectively, from units (3,1) to (3,10). Similarly, we observed that the Estrada index of BiI3 and BRE was 0.00515 and 0.0523, respectively, from units (3,1) to (3,10). We have used two other methods to support our results. In the first method, we have found the relative error percentage (%) and in the second method, we have found the average absolute error and the standard deviation along with their normal curve. In the first method, the relative error percentage (%) was less than 0.1 %, which suggested that this method was accurate for this dataset, with errors typically less than 0.1 % relative to the exact values. In the second method, a small value of standard deviation indicated that the data points were very close to the mean, reflecting low variability, high consistency, tight distribution, and predictability.

For future research, we propose to conduct comparative studies with other data-driven and other machine learning approaches, such as neural networks, SVM, and decision trees, to evaluate the relative performance and applicability of different methods. Apply the polynomial curve fitting method to a wider range of molecular structures and chemical systems to test its generalizability and robustness. Explore the integration of more advanced machine learning techniques to improve prediction accuracy and computational efficiency. Investigate the use of our methodology in real-world applications, such as drug discovery, materials science, and chemical engineering, to assess its practical utility and impact. Develop a more comprehensive framework that combines various data-driven approaches for a holistic understanding and prediction of molecular properties. The error analysis method we used can be applied to any model. Relative error percentage is scale-independent, making it useful for comparing errors across different datasets or models. It provides a direct interpretation of how large the errors are relative to the actual values, making it easier to understand the model's performance in practical terms. Standard deviation offers a well-rounded understanding of the model's performance, highlighting different aspects of accuracy and consistency, which can be crucial for model evaluation and improvement.

## Funding

This research was funded by Deputyship for Research and Innovation, 10.13039/100009950Ministry of Education in Saudi Arabia through the project number ISP-2024.

## Data availability statement

No external data is used, all the data required to understand, evaluate, and reproduce the findings presented in this manuscript are included in the manuscript.

## CRediT authorship contribution statement

**Ibtisam Masmali:** Writing – review & editing, Data curation, Conceptualization. **Muhammad Faisal Nadeem:** Writing – review & editing, Writing – original draft, Validation, Methodology, Investigation, Data curation, Conceptualization. **Zeeshan Saleem Mufti:** Writing – review & editing, Writing – original draft, Software, Methodology, Formal analysis. **Ali Ahmad:** Writing – review & editing, Validation, Software, Methodology, Data curation. **Ali N.A. Koam:** Writing – review & editing, Validation, Investigation. **Haleemah Ghazwani:** Writing – review & editing, Validation, Software.

## Declaration of competing interest

The authors declare that they have no known competing financial interests or personal relationships that could have appeared to influence the work reported in this paper.

## References

[1] Schüler M., Pavlyukh Y. (2018). Spectral properties from Matsubara Green's function approach: application to molecules. Phys. Rev. B.

[2] Manivel S., Gangadharappa B.S., Elangovan N., Thomas R., Ali O.A.A., Saleh D.I. (2022). Schiff base (Z)-4-((furan-2-ylmethylene) amino) benzenesulfonamide: synthesis, solvent interactions through hydrogen bond, structural and spectral properties, quantum chemical modeling and biological studies. J. Mol. Liq..

[3] Hołaj-Krzak J.T., Rekik N., Alsaif N.A., Lakshminarayana G. (2022). Elucidating the infrared spectral properties of succinic molecular acid crystals: illustration of the structure and the hydrogen bond energies of the crystal and its deuterated analogs. J. Phys. Chem..

[4] Langhoff P.W., Hinde R.J., Boatz J.A., Sheehy J.A. (2002). Spectral theory of the chemical bond. Chem. Phys. Lett..

[5] Gribov L.A., Baranov V.I., Mikhailov I.V. (2023). In Advances in Geochemistry, Analytical Chemistry, and Planetary Sciences: 75th Anniversary of the Vernadsky Institute of the Russian Academy of Sciences.

[6] Choudhary V., Bhatt A., Dash D., Sharma N. (2019). DFT calculations on molecular structures, HOMO–LUMO study, reactivity descriptors and spectral analyses of newly synthesized diorganotin (IV) 2-chloridophenylacetohydroxamate complexes. J. Comput. Chem..

[7] Keith J.A., Vassilev-Galindo V., Cheng B., Chmiela S., Gastegger M., Müller K.R. (2021). Tkatchenko A. Combining machine learning and computational chemistry for predictive insights into chemical systems. Chem. Rev..

[8] Strieth-Kalthoff F., Sandfort F., Segler M.H., Glorius F. (2020). Machine learning the ropes: principles, applications and directions in synthetic chemistry. Chem. Soc. Rev..

[9] Artrith N., Butler K.T., Coudert F.X., Han S., Isayev O., Jain A., Walsh A. (2021). Best practices in machine learning for chemistry. Nat. Chem..

[10] Oviedo F., Ferres J.L., Buonassisi T., Butler K.T. (2022). Interpretable and explainable machine learning for materials science and chemistry. Acc. Mater. Res..

[11] Moosavi S.M., Jablonka K.M., Smit B. (2020). The role of machine learning in the understanding and design of materials. J. Am. Chem. Soc..

[12] Jiao Z., Hu P., Xu H., Wang Q. (2020). Machine learning and deep learning in chemical health and safety: a systematic review of techniques and applications. ACS Chemical Health & Safety.

[13] Haghighatlari M., Hachmann J. (2019). Advances of machine learning in molecular modeling and simulation. Current Opinion in Chemical Engineering.

[14] Liebal U.W., Phan A.N., Sudhakar M., Raman K., Blank L.M. (2020). Machine learning applications for mass spectrometry-based metabolomics. Metabolites.

[15] Hyde K.K., Novack M.N., LaHaye N., Parlett-Pelleriti C., Anden R., Dixon D.R., Linstead E. (2019). Applications of supervised machine learning in autism spectrum disorder research: a review. Review Journal of Autism and Developmental Disorders.

[16] Mao Y., Dong N., Wang L., Chen X., Wang H., Wang Z., Kislyakov I.M., Wang J. (2020). Machine learning analysis of Raman spectra of MoS2. Nanomaterials.

[17] Boes J.R., Mamun O., Winther K., Bligaard T. (2019). Graph theory approach to high-throughput surface adsorption structure generation. J. Phys. Chem..

[18] Balaban A.T. (1985). Applications of graph theory in chemistry. J. Chem. Inf. Comput. Sci..

[19] Burch K.J. (2019). Chemical Applications of Graph Theory.

[20] Hall G.G. (1977). On the eigenvalues of molecular graphs. Mol. Phys..

[21] Gutman I., Kiani D., Mirzakhah M. (2009). On incidence energy of graphs. MATCH Communications in Mathematical and in Computer Chemistry.

[22] Chen Y., Zhu J. (2019). A graph theory-based method for regional integrated energy network planning: a case study of a China–US low-carbon demonstration city. Energies.

[23] Bozkurt S.B., Gutman I. (2013). Estimating the incidence energy. MATCH Communications in Mathematical and in Computer Chemistry.

[24] Das K.C., Gutman I. (2014). On incidence energy of graphs. Lin. Algebra Appl..

[25] Estrada E. (2000). Characterization of 3D molecular structure. Chem. Phys. Lett..

[26] Malik M.A., Farooq R. (2015). Computational results on the energy and Estrada index of TUC4C8 (R)[m, n] nanotubes. Optoelectronics and Advanced Materials-Rapid Communications.

[27] Omidi G.R. (2009). On the nullity of bipartite graphs. Graph. Combinator.

[28] Imran M., Ali M.A., Ahmad S., Siddiqui M.K., Baig A.Q. (2018). Topological characterization of the symmetrical structure of bismuth tri-iodide. Symmetry.

[29] Mackay R.A., Henderson W. (2002 Nov 18).

[30] Smart L.E., Moore E.A. (2012 May 29). Solid state chemistry: an introduction. CRC press.

[31] Rebouillat S., Pla F. (2016). Recent strategies for the development of biosourced-monomers, oligomers and polymers-based materials: a review with an innovation and a bigger data focus. J. Biomaterials Nanobiotechnol..

[32] Brandt R.E., Kurchin R.C., Hoye R.L., Poindexter J.R., Wilson M.W., Sulekar S., Lenahan F., Yen P.X., Stevanovic V., Nino J.C., Bawendi M.G. (2015). Investigation of bismuth triiodide (BiI3) for photovoltaic applications. J. Phys. Chem. Lett..

[33] Sankaran S., Deshmukh K., Ahamed M.B., Pasha S.K. (2019). In Polymer Nanocomposites in Biomedical Engineering.

[34] Lintereur AT, Qiu W, Nino JC, Baciak JE. Bismuth tri-iodide radiation detector development. InHard X-Ray, Gamma-Ray, and Neutron Detector Physics XI 2009 Sep 17 (Vol. vol. 7449, p. 74491M). International Society for Optics and Photonics.

[35] Tongay S., Fan W., Kang J., Park J., Koldemir U., Suh J., Narang D.S., Liu K., Ji J., Li J., Sinclair R. (2014). Tuning interlayer coupling in large-area heterostructures with CVD-grown MoS2 and WS2 monolayers. Nano Lett..

[36] Xie C., Yan F. (2017). Flexible photodetectors based on novel functional materials. Small.

[37] Matsumoto M., Hitomi K., Shoji T., Hiratate Y. (2002). Bismuth tri-iodide crystal for nuclear radiation detectors. IEEE Trans. Nucl. Sci..

[38] Gokhale S.S., Han H., Baciak J.E., Nino J.C., Jordan K.A. (2015). Growth, fabrication, and testing of bismuth tri-iodide semiconductor radiation detectors. Radiat. Meas..

[39] Brandt R.E., Kurchin R.C., Hoye R.L., Poindexter J.R., Wilson M.W., Sulekar S., Lenahan F., Yen P.X., Stevanovic V., Nino J.C., Bawendi M.G. (2015). Investigation of bismuth triiodide (BiI3) for photovoltaic applications. J. Phys. Chem. Lett..

